# Parent Perceptions of Telemedicine for Acute Pediatric Respiratory Tract Infections: Sequential Mixed Methods Study

**DOI:** 10.2196/49170

**Published:** 2024-01-16

**Authors:** Sarah K Burns, Tamar Krishnamurti, Tran T Doan, Janel Hanmer, Alejandro Hoberman, Jeremy M Kahn, Kelsey Schweiberger, Kristin N Ray

**Affiliations:** 1 Department of Pediatrics University of Pittsburgh School of Medicine Pittsburgh, PA United States; 2 Department of Medicine University Pittsburgh School of Medicine Pittsburgh, PA United States; 3 Department of Health Policy & Management University of Pittsburgh Graduate School of Public Health Pittsburgh, PA United States; 4 Department of Critical Care Medicine University of Pittsburgh School of Medicine Pittsburgh, PA United States

**Keywords:** telemedicine, telehealth, acute care, acute, pediatrics, pediatric, family medicine, family-centered, child, children, parent, parents, attitude, attitudes, opinion, perception, perceptions, perspective, perspectives, expectation, expectations

## Abstract

**Background:**

Since 2020, parents have had increasing opportunities to use telemedicine for their children, but how parents decide whether to use telemedicine for acute pediatric care relative to alternative sites of care is not clear. One of the most common reasons parents seek acute care for their children is for acute respiratory tract infections (ARTIs).

**Objective:**

This study aims to examine parental expectations of care via telemedicine for pediatric ARTIs, contrasting expectations of care delivered via primary care telemedicine and direct-to-consumer (DTC) telemedicine.

**Methods:**

We performed a sequential mixed methods analysis to examine how parents assess telemedicine for their children’s acute care. We used ARTIs as a case study for examining parent perceptions of telemedicine. First, we analyzed semistructured interviews focused on parent responses about the use of telemedicine. Each factor discussed by parents was coded to reflect whether parents indicated it incentivized or disincentivized their preferences for telemedicine versus in-person care. Results were organized by a 7-dimension framework of parental health care seeking that was generated previously, which included dimensions related to care sites (expected access, affordability, clinical quality, and site quality) and dimensions related to child or family factors (perceived illness severity, perceived child susceptibility, and parent self-efficacy). Second, we analyzed responses to a national survey, which inquired about parental expectations of primary care telemedicine, commercial DTC telemedicine, and 3 in-person sites of care (primary care, urgent care, and emergency department) across 21 factors identified through prior qualitative work. To assess whether parents had different expectations of different telemedicine models, we compared survey responses for primary care telemedicine and commercial DTC telemedicine using weighted logistic regression.

**Results:**

Interview participants (n=40) described factors affecting their perceptions of telemedicine as a care modality for pediatric ARTIs. Generally, factors aligned with access and affordability (eg, decreased wait time and lower out-of-pocket cost) were discussed as potential incentives for telemedicine use, while factors aligned with perceived illness severity, child susceptibility, and clinician quality (eg, trustworthiness) were discussed as potential disincentives for telemedicine use. In survey responses (n=1206), primary care and commercial DTC telemedicine were rated similarly on items related to expected accessibility and affordability. In contrast, on items related to expected quality of care, primary care telemedicine was viewed similarly to in-person primary care, while commercial DTC telemedicine was rated lower. For example, 69.7% (weighted; 842/1197) of respondents anticipated their children would be comfortable and cooperative with primary care telemedicine versus 49.7% (weighted; 584/1193) with commercial DTC telemedicine (*P*<.001).

**Conclusions:**

In a mixed methods analysis focused on telemedicine for ARTIs, parents expressed more concerns about telemedicine quality in commercial DTC models compared with primary care–based telemedicine. These results could help health systems better design telemedicine initiatives to support family-centered care.

## Introduction

The decision to seek care for a sick child is an increasingly complex one for parents. Parents are faced with a variety of care site options, which include both in-person (ie, primary care, urgent care, and emergency department [ED]) and virtual care modalities (ie, primary care provider [PCP] telemedicine and commercial direct-to-consumer [DTC] telemedicine). In recent years, families have increased experience and opportunity to use telemedicine with both their PCP and DTC telemedicine companies [1-4]. Many studies of prior telemedicine users indicate high parent satisfaction after telemedicine use, including high satisfaction with interpersonal and technical components of these visits, while others note concerns about the quality of interpersonal interaction and concerns about misdiagnosis and privacy [5-8]. Studies of parent perceptions as they anticipate or make decisions about potential telemedicine use are fewer. Some have importantly detailed disparities in general willingness to use telemedicine by sociodemographic characteristics or technology ownership [9,10]. Beyond general willingness to use telemedicine, however, it is important to understand decision factors influencing parents toward or away from telemedicine use at the time of a specific health care need.

One common reason for problem-based visits among children is the cluster of diagnoses known as acute respiratory tract infections (ARTIs), which include viral (eg, viral upper respiratory infection and viral pharyngitis) and bacterial infections (eg, streptococcal pharyngitis and acute otitis media). Before the pandemic, ARTIs accounted for over one-third of acute pediatric primary care visits and nearly 50% of DTC telemedicine visits [4,11]. The volume of pediatric ARTI visits dropped substantially during the early pandemic but increased over time with otitis media, streptococcal sore throat, and acute upper respiratory infection remaining common acute visit diagnoses for both in-person and telemedicine care during the later pandemic period [2,12-15]. These data suggest that deciding whether to seek care for pediatric cough and cold symptoms is one of the more common care-seeking decisions faced by parents. Thus, we focus on ARTIs as an illustrative example of a reason parents may seek care for their child, providing an opportunity to examine care-seeking decision-making processes. Important prior studies uncovered factors that may influence family decision-making about ARTI care-seeking, including site accessibility (eg, timeliness and geographic) and quality (eg, interpersonal and clinical) as well as illness and family factors [16-18], but did not explore how the option of telemedicine informs these decisions. More recent qualitative studies have begun to explore ARTI care-seeking decisions in the context of the option of telemedicine [19,20]. As an example, we recently examined parents decision-making for pediatric ARTIs in the context of the option of telemedicine and identified 7 dimensions that influence parents’ decisions: perceived illness severity, perceived child susceptibility, parental self-efficacy, expected accessibility of care, expected affordability of care, expected quality of clinician, and expected quality of site [19]. By applying this framework to qualitative and quantitative data focused specifically on parent perceptions of the potential values and risks of telemedicine use, we seek now to understand how families assess individual factors to decide whether to use telemedicine for a specific ARTI care need. Understanding parent perceptions of telemedicine across these 7 dimensions is important to support health care systems in incorporating telemedicine into the acute care landscape beyond the public health emergency.

Therefore, in this analysis, we investigated current parent views regarding decision factors that potentially influence their intention to use telemedicine and other sites of acute care when their children have ARTI symptoms. Specifically, we sought to better understand parent perceptions of telemedicine models compared with in-person care for pediatric ARTIs using both qualitative and quantitative data to specifically examine potential differences in expectations of telemedicine delivered via PCPs versus telemedicine delivered via commercial DTC providers.

## Methods

### Overview

We performed a sequential mixed methods analysis to examine how parents approach decisions about the use of telemedicine when care-seeking for commonly experienced acute illness. We first conducted a qualitative analysis of semistructured parent interviews in which we asked parents to discuss prior care-seeking when their children were experiencing ARTI symptoms. Previous analysis of specific portions of these semistructured interviews which focused broadly on the decision to seek care published previously reported on 7 broad dimensions that parents consider when deciding whether and where to seek care for their children (perceived illness severity, perceived child susceptibility, parental self-efficacy, expected accessibility of care, expected affordability of care, expected quality of clinician, and expected quality of site) [19]. In this analysis, we examined parent responses to a specific later portion of the interviews where parents were asked to reflect specifically on the option to use telemedicine, which we analyzed to elucidate factors that might incentivize or disincentivize parents to seek ARTI care through telemedicine. Building on these qualitative findings, we fielded and analyzed a national survey to examine pediatric telemedicine use. Prior analysis examined parent-reported use of primary care telemedicine [21]. In this analysis, we focus instead on parent expectations of the care their child would receive if they presented at each of 5 different sites of care: in-person primary care, in-person urgent care, in-person ED, PCP telemedicine, and commercial DTC telemedicine. We adhered to the standards for reporting quality research guidelines for reporting the qualitative portion of this research [22].

### Semistructured Interviews

As described in greater detail elsewhere, we first interviewed 16 pediatric research and clinical professionals to establish a normative expert model of parent care-seeking. Insights from these interviews then informed an interview guide for subsequent semistructured interviews with parents. Prior to launching interviews with a full sample (n=40), we conducted 3 pilot interviews with parents to ensure clarity of our questions and definitions. The first portion of the open-ended interview guide inquired about perceived needs, desires, and care-seeking decisions when seeking care generally for a child’s ARTI, with results reported previously [19]. This paper focuses on a portion of the interview guide that inquired about the risks and benefits of telemedicine care for a child’s ARTI (“What are your thoughts on the benefits of having a provider see your child while you are in your own house?” and “When, if ever, would telemedicine feel like a good choice for a child with a cold?”) and barriers to telemedicine use (“What might make a telemedicine visit difficult for your family?”). Parents of children aged 1-5 years were recruited through a research registry of parents in Western Pennsylvania and beyond. All interviews were recorded, transcribed, and analyzed using thematic content analysis. Team members (SB, KR, and TK) independently coded each sentence in the first 5 transcripts and together reviewed and then developed a preliminary codebook of a priori and emergent codes. For each code present, we noted, when relevant, whether the factor was discussed specifically as a reason to use or avoid telemedicine. Upon achieving consensus, a codebook containing definitions and rules was finalized. The remaining transcripts were coded by a primary coder (SB), and 14 of those were cocoded by a second coder (KR or TK). Dedoose (SocioCultural Research Consultants), a qualitative research software program, was used to code interviews. In this paper, we present parental perceptions of potential factors that might positively or negatively influence their interest in the use of telemedicine organized by the previously identified 7 dimensions [19] to illustrate the degree to which these dimensions influence parental decisions to seek care from telemedicine.

### National Survey

As described in greater detail elsewhere [21], we then developed a survey informed by our prior qualitative findings and fielded the survey nationally through the University of Chicago’s AmeriSpeak Panel [23], a nationally representative panel. The survey included items asking about parent priorities and expectations when seeking care for a child’s ARTI, prior telemedicine use, and sociodemographic characteristics. The survey underwent cognitive testing with 3 parents of young children and was offered in English and Spanish. Prior analysis of survey data focused on parent-report of PCP telemedicine use relative to sociodemographic characteristics of the respondent [21]. This analysis focuses on parent expectations of 21 different factors with the potential to influence their care-seeking across 5 different sites of care: in-person primary care, in-person urgent care, in-person ED, PCP telemedicine, and commercial DTC telemedicine. The order in which care sites were presented to respondents was randomized.

In the survey, PCP telemedicine was described as “a telemedicine visit with your child’s usual primary care office or clinic. This would be a virtual visit with the provider or group of providers that conduct in-person well and sick care for your child(ren) in the office or clinic.” DTC commercial telemedicine was described as “a telemedicine company or group that focuses on telemedicine visits rather than in-person care (also called DTC telemedicine). Providers in these groups do not provide care in-person and are not part of your child’s usual care team. In these visits, you connect online and see an available provider in a model that could be thought of as virtual urgent care. Some DTC telemedicine groups or companies are affiliated with health systems but are still separate from primary care clinics who might see their own patients through telemedicine.”

The survey was fielded to members of the AmeriSpeak panel, with panel members eligible if they were caregivers of children aged ≤17 years.

This analysis focused on the percentage of respondents who anticipated that the specified care site would meet their expectations “always” or “often” for each of the 21 specific items. For each of the 21 items, we calculated the weighted percentage of respondents who indicated they “always” or “often” expect that item at each site, using weights derived from panel sampling weights along with survey response rates, such that the demographics of the weighted sample align with the US Current Population Survey [24]. Surveys with missing responses to individual items were omitted from denominator for that item. We then used *t* statistics from weighted logistic regression models to compare responses for each item for PCP telemedicine and DTC telemedicine. Finally, we averaged responses to items within each of the 7 dimensions for PCP telemedicine and DTC telemedicine to further synthesize the differences between expectations of PCP and DTC telemedicine.

### Ethical Considerations

#### Semistructured Interviews

The qualitative interview portion of this study was determined exempt from human participants review by the University of Pittsburgh institutional review board (IRB; STUDY20040025). Participants received an IRB-approved introductory script prior to participating in the interview informing them of the goals of the interview, potential risks and benefits, plans to protect their information, and how to contact the research team if they had questions, and they then provided verbal consent to proceed. Identifiable data collected only for payment purposes were stored separately from interview data; interview data were deidentified. Participants received a US $50 gift card through the University of Pittsburgh’s Vincent Payment Solutions.

#### National Survey

The survey portion of this study was determined exempt from human participants review by the University of Pittsburgh IRB and by the National Opinion Research Center at the University of Chicago IRB (STUDY21070080). The University of Pittsburgh research team only received deidentified data from National Opinion Research Center for analysis. Survey respondents were compensated for their time through AmeriSpeak, receiving the cash equivalent of $5 (equivalent of US $17.50/hour) for completing the survey.

## Results

### Qualitative Interview Results

A total of 40 parents participated in the qualitative interviews, of which 65% (n=26) of parents had more than 1 child, 65% (n=26) of parents had previous experience using telemedicine for a child, and 38% (n=15) of parents had children insured by a commercial or employer-sponsored insurer (Table 1).

**Table 1:** Demographic characteristics of participants in qualitative parent interviews (n=40).

Characteristics			Interview participants, n (%)^a^
**Sex**			
	Female	38 (95)	
	Male	2 (5)	
**Age group (years)**			
	18-30	12 (30)	
	31-40	23 (58)	
	41-50	5 (13)	
**Interviewee’s children’s ages^b^**			
	Interviewees with children <1 year	5 (13)	
	Interviewees with children 1-5 year	40 (100)	
	Interviewees with children >5 year	16 (40)	
**Self-reported race** **and ethnicity**			
	African American or Black	6 (15)	
	African American and Native American	1 (3)	
	Hispanic or Hispanic and multiracial	3 (8)	
	White	30 (75)	
**Prior telemedicine use for their child**			
	No	14 (35)	
	Yes	26 (65)	
**Geographic location**			
	Urban	23 (58)	
	Rural	17 (42)	
**Insurance type**			
	Commercial or employer based	15 (38)	
	Medicaid or federal	25 (63)	

^a^Percentages may sum to >100% due to rounding.

^b^Categories are not mutually exclusive.

### Dimensions Affecting Parental Perceptions of Telemedicine

Interviewees described a range of factors that affect their perceptions of telemedicine care for their children. These factors mapped onto the 7 dimensions influencing care-seeking, including dimensions related to care sites (expected access, affordability, clinical quality, and site quality) and dimensions related to child or family factors (perceived illness severity, perceived child susceptibility, parent self-efficacy) [19].

#### Expected Accessibility of the Site

Interviewees expressed interest in using telemedicine due to perceived opportunities to increase temporal accessibility, mitigate geographic accessibility, and maximize convenience (Multimedia Appendix 1). In contrast, some spoke about the value of telemedicine only as a last resort option when other sites could not be accessed. One interviewee described how using telemedicine maximizes convenience as follows: “I honestly couldn’t see how [using telemedicine] would make anything difficult, ‘cause it’s saving me time, gas, all of that stuff” (parent 24). In contrast, some interviewees expressed concerns about digital accessibility and how telemedicine might be less optimal for their family or for other families: “You might not even have access to computer. I mean I guess everybody has a cell phone, but, you know, connecting to like a video call on your cell phone isn’t always ideal” (parent 06).

#### Expected Affordability of the Site

Interviewees favored telemedicine use if out-of-pocket costs were less than or equal to in-person options: “I mean you’re not getting that one-on-one or face-to-face time necessarily, so, I kind of feel like [telemedicine] should probably be cheaper [laughter] or free” (parent 06). Parents viewed telemedicine favorably if insurance would cover the expense and less favorably if there was a likelihood of needing further in-person evaluation contributing to a possible second visit expense or when costs for telemedicine were out-of-pocket.

#### Expected Quality of the Site

Interviewees viewed telemedicine positively when they perceived that telemedicine increased their child’s comfort level and made seeking care safer: “Well, the kids will be more comfortable [on telemedicine]. I mean, ‘cause I know kids...they get anxious when they go to the doctor’s office, so they’re symptoms might get worse...I mean, they might also pick other stuff up when they go to the doctor’s office. So it might just be an ease, so you’re not exposing yourself to other things” (parent 39). Parents were split on whether they felt like telemedicine would allow for adequate assessment of ARTI symptoms, noting that evaluation of some symptoms (eg, ear pain) might be more difficult than others (eg, red eyes) over telemedicine without the availability of equipment with remote assessment capabilities. In contrast, some parents were less receptive to the idea of seeking telemedicine, and pointed out that a telemedicine appointment cannot provide comprehensive clinical care: “It’s kind of hard—you can’t really do immunization [on telemedicine]—I mean, ‘cause you would still have to go to the office to get those” (parent 39).

#### Expected Quality of the Clinician

Interviewees discussed intersections between telemedicine and the expected quality of clinicians. Some interviewees indicated they would be less interested in using telemedicine if they could not visit a familiar provider: “If it was doctor that...didn’t know [my child] well, I might not feel 100% comfortable. But because [pediatrician] knows him and his personality...I’d probably feel more comfortable if [telemedicine] was with her” (parent 12). Interviewees were divided on whether they perceived they would receive reassurance over telemedicine. There was a general perception among interviewees that receiving trustworthy care would be less likely over telemedicine. Additionally, parents expressed more interest in telemedicine when the provider was someone with experience in caring for children. One parent described their preference for a provider with pediatric expertise: “I don’t want, you know, like, a doctor who just got their degree last week to try and diagnose what my son has, like, with his cold and everything. I would want someone looking at it who has experience with kids” (parent 31).

#### Perceived Severity of Illness of the Child

Parent interviewees discussed perceptions of telemedicine that mapped along the following 3 primary child and parent dimensions: perceived severity, perceived child susceptibility, and parental self-efficacy (Multimedia Appendix 2). Interviewees primarily viewed telemedicine less favorably when they perceived the high severity of a child’s illness and symptom complexity. One interviewee described her care-seeking decision when her child had a cold: “I think if it’s just like a normal cold...I feel like I would be pretty comfortable doing telemedicine for that. And then if they thought it was severe enough, then I would go in” (parent 30). Parent’s perceptions of using telemedicine based on their child’s demeanor and appearance were divided, with interviewees expressing both interest and disinterest in using telemedicine when their child appeared more ill. In contrast, interviewees generally viewed the use of telemedicine favorably when prolonged persistence of their child’s symptoms was the primary driver of care seeking.

#### Perceived Susceptibility of the Child

Interviewees generally expressed less interest in telemedicine use for ARTI acute care when they perceived greater underlying susceptibility of the child, such as if they have a child with medical complexity or younger age. One interviewee described her preference for in-person care because of a perceived vulnerability to the illness of her child: “I need my child to be seen by somebody because I need them to listen to her lungs, and I need them to check her ears. Maybe if the child is not prone to having ear infections, and she’s not asthmatic, then it would be a little different” (parent 33). In contrast, interviewees showed more interest in telemedicine use when trying to avoid community-based exposure: “I’m thinking now I probably should have done [telemedicine] instead of having to take him in, and like possibly exposing him” (parent 12).

#### Perceived Self-Efficacy of the Parent

Parent self-efficacy factors identified by interviewees included achieving the goal of the visit, antibiotic expectations, and easing uncertainty, all of which interviewees generally viewed as achievable through telemedicine care. For individual interviewees, however, self-efficacy factors, such as parent health literacy and worry, were discussed as individual reasons to seek and not to seek care through telemedicine. Concerns about the ability to protect their child’s privacy (ie, information privacy and physical location privacy) and negotiate power differentials (ie, equity and patient-clinician power dynamics) contributed to parental worry about using telemedicine. One parent described these worries: “They [provider on telemedicine] could catch you at a really bad time whenever, you know, ‘cause when a kid is sick...things in the house just kind of—everything falls into chaos, so they could be seeing a snapshot and judging your entire life by that” (parent 03).

### Survey Results

Survey invitations were sent to 6015 AmeriSpeak panelists, with 1599 (26.6%) of those invited completing the screener; of the 1599 individuals screened, 1297 (81.1%) met the eligibility requirements; and of the 1297 individuals who were eligible, 1206 (93.0%) completed the survey. The majority (1136/1206; 96% weighted) of respondents took the survey in English, and 60% (weighted; 714/1206) had children insured through a private employer or purchased directly (Table 2).

**Table 2:** Demographic characteristics of participants (n=1206) and weighted percentages in a quantitative national survey.

Characteristics		Survey respondents, n (weighted %)^a^
**Sex**		
	Female	786 (55.3)
	Male	420 (44.7)
**Age group (years)**		
	18-29	158 (11.8)
	30-44	761 (59.2)
	45-59	258 (26.3)
	>60	29 (2.6)
**Race** **and ethnicity**		
	Asian, non-Hispanic	34 (6.4)
	Black, non-Hispanic	109 (11.3)
	Hispanic	375 (22)
	White, non-Hispanic	634 (56.6)
	Other, non-Hispanic	54 (3.7)
**Census division**		
	South Atlantic	233 (19.5)
	Pacific	195 (16.7)
	East North Central	172 (14.3)
	West South Central	136 (13.2)
	Mountain	129 (8)
	Mid-Atlantic	103 (11.4)
	West North Central	102 (6.6)
	East South Central	77 (6)
	New England	59 (4.4)
**Survey language**		
	English	1136 (96.1)
	Spanish	70 (3.9)
**Previous telemedicine use for a child**		
	Yes	516 (41)
**Child insurance type**		
	Employer or Commercial	714 (60.2)
	Medicaid or federal	460 (37)
	Uninsured	32 (2.8)

^a^Percentages may sum to >100% due to rounding.

Parents were asked to indicate how often they expect to find each of the 21 specific items across 5 different care sites. For most items, respondents most commonly expected to experience that item at an in-person primary care visit compared with the 4 other sites. The 2 virtual sites of care carried higher expectations than in-person primary care for items related to accessibility and not being near other sick children (Multimedia Appendix 3). For example, out of 1200 respondents answering the item, 56.3% (weighted; n=679) expected to avoid a long wait through a primary care visit, compared with 60% (weighted; 740/1195) through commercial DTC telemedicine and 64.1% weighted (790/1200) through PCP telemedicine. The 2 virtual sites carried lower expectations than all 3 in-person sites for “being able to complete all tasks” (within the expected quality of site dimension) and “ability to care for severe symptoms” (within the perceived illness severity dimension; Table 3 and Multimedia Appendix 3).

Parents reported high expectations for PCP telemedicine across several system dimensions (Table 3), with three-quarters indicating expecting to be able to usually or always see a provider with experience caring for children (919/1195; 76.5% weighted) and to receive care in a way that protects the child’s privacy (932/1198; 76.2% weighted). Parents largely had higher expectations for PCP telemedicine than commercial DTC telemedicine for items related specifically to their perception of their child’s illness and susceptibility with 75.7% (weighted; 915/1198) always or often expecting the ability to receive care across the 0-17 years age range at PCP telemedicine (Table 4 and Multimedia Appendix 4).

Comparing expectations specifically for the 2 telemedicine options, responses were relatively similar for accessibility items and were the most discrepant for quality of clinician and child susceptibility items. For example, a similar percentage of parents expected to always or often receive care that does not disrupt their schedule for PCP telemedicine (723/1200; 60.2% weighted) and DTC telemedicine (696/1195; 59.1% weighted; *P*=.57), which is an item under the accessibility dimension. In contrast, parents’ expectations to often or usually receive care from a “provider who they trust to make choices in their child’s best interest” varied from 72.4% (weighted; 885/1199) for PCP telemedicine to 54.3% (weighted; 664/1192) for DTC telemedicine (*P*<.001). Similarly, parents’ expectations to always or often receive care from a “provider with full access to their child’s medical history” ranged from 71.3% (weighted; 857/1194) for PCP telemedicine to 46.5% (weighted; 541/1190) for DTC telemedicine (*P*<.001).

**Figure 1:**
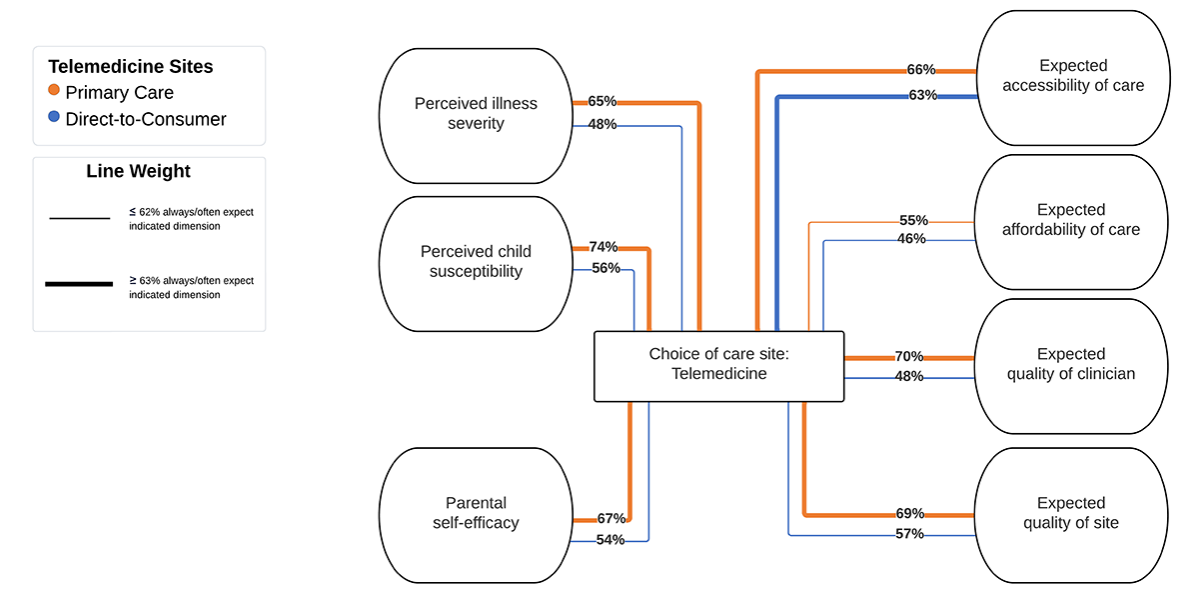
Percent of parents expecting each of 7 dimensions of the influence diagram of health care–seeking decisions when seeking care at primary care provider telemedicine and direct-to-consumer telemedicine. The center rectangle represents the decision “choice of care site.” Focusing on the choice to use either of the 2 studied telemedicine models (primary care telemedicine and commercial direct-to-consumer telemedicine), this choice is surrounded by dimensions affecting this decision in ovals with averaged expectations of survey respondents of primary care telemedicine (orange) and direct-to-consumer telemedicine (blue). Line weight indicates the average percentage of respondents expecting the factors within the indicated dimension always or often at the indicated telemedicine site.

Survey results were averaged within each of the 7 dimensions and mapped onto the previously identified health care–seeking decision model (Figure 1), with line weight illustrating the difference in parent expectations of PCP and DTC telemedicine.

**Table 3:** Percentage of caregiver respondents (n=1206) who “always” or “often” expect the factors listed relating to expected accessibility, affordability, and quality at the 2 telemedicine sites (primary care provider [PCP] telemedicine and direct-to-consumer [DTC] telemedicine).

Factors^a^		PCP telemedicine, n/N (weighted %)	DTC telemedicine, n/N (weighted %)	*P* value
**Expected accessibility**				
	Fits schedule	723/1200 (60.2)	696/1195 (59.1)	.57
	Minimal hassle	892/1199 (73.9)	843/1195 (70.4)	.045
	Minimal wait	790/1200 (64.1)	740/1195 (60.0)	.04
**Expected affordability**				
	Minimal costs	676/1197 (54.5)	553/1196 (46.2)	<.001
**Expected quality of the site**				
	Knows child	726/1198 (60.5)	416/1196 (35.9)	<.001
	Pediatric experience	919/1195 (76.5)	672/1193 (55.9)	<.001
	Record access	857/1194 (71.3)	541/1190 (46.5)	<.001
	Can trust	885/1199 (72.4)	664/1192 (54.3)	<.001
**Expected quality of the site**				
	Protects privacy	932/1198 (76.2)	756/1196 (61.8)	<.001
	Comprehensive tasks	656/1194 (56.1)	505/1196 (42.3)	<.001
	Avoid exposures	890/1197 (73.4)	856/1192 (72.4)	.54
	Child comfort	842/1197 (70.3)	584/1193 (50.2)	<.001

^a^For each item, we calculated the weighted percentage of nonmissing responses indicating that the item was expected “always” or “often” at the specified site. We determined statistical significance using *t* statistics from weighted logistic regression models to compare responses for each item for PCP telemedicine and DTC telemedicine.

**Table 4:** Percentage of caregiver respondents (n=1206) who “always” or “often” expect the factors listed relating to perceived illness severity, perceived child susceptibility and parental self-efficacy factors at the two telemedicine sites (primary care provider [PCP] telemedicine and direct-to-consumer [DTC] telemedicine).

Factors^a^			PCP telemedicine, n/N (weighted %)		DTC telemedicine, n/N (weighted %)	*P* value
**Perceived illness severity**						
	Severity responsive	773/1199 (63.1)		585/1198 (48.3)		<.001
	Duration responsive	792/1196 (65.3)		576/1194 (48.1)		<.001
	Multiple symptom responsive	814/1199 (67.9)		634/1198 (51.6)		<.001
	Seriousness responsive	762/1199 (63.3)		553/1196 (46.0)		<.001
	Mood responsive	790/1196 (64.4)		579/1190 (47.6)		<.001
**Perceived child susceptibility**						
	Considers history	902/1193 (73.1)		646/1195 (52.6)		<.001
	Age responsive	915/1198 (75.7)		718/1193 (58.4)		<.001
**Parental self-efficacy**						
	Will understand	892/1200 (73.1)		703/1198 (57.9)		<.001
	Obtain doctor forms	754/1199 (61.8)		609/1198 (50.6)		<.001

^a^For each item, we calculated the weighted percentage of nonmissing responses indicating that the item was expected “always” or “often” at the specified site. We determined statistical significance using *t* statistics from weighted logistic regression models to compare responses for each item for PCP telemedicine and DTC telemedicine.

## Discussion

Through semistructured interviews, parents expressed positive assessments of telemedicine accessibility but voiced concerns about telemedicine quality. In a structured survey informed by these results, we found continued positive expectations of telemedicine accessibility and less concern about quality for PCP telemedicine relative to commercial DTC telemedicine. Respondents anticipated that both models of telemedicine would minimize hassle, wait time, schedule disruption, and exposure to other ill children. However, respondents had higher expectations of clinician and site quality and the ability to treat severe illness from PCP telemedicine compared with DTC telemedicine. The strengths of this analysis include a mixed methods approach with qualitative interviews conducted until saturation was achieved followed by a large nationally representative survey, which was conducted 2 years into the COVID-19 pandemic.

These findings first provide an illustration of the applicability of our health care–seeking decision model [19], which incorporates elements represented in prior models representing both health beliefs and access to care [25,26]. By using the health care–seeking decision model as a guiding framework for the interpretation of these qualitative and quantitative data, we uncovered differences in parent perceptions of expected quality versus expected accessibility across different models of telemedicine care. The survey data further support the health care–seeking decision model, with these quantitative items showing variation across sites and dimensions.

The relatively high expectations of quality in PCP telemedicine—and the contrast to expectations in DTC telemedicine—comes at an important time for state and federal policy makers as the COVID-19 public health emergency has ended. These findings indicate that parents differentiate between primary care models of telemedicine and virtual-only telemedicine, but this is not a distinction that has made its way into all payment and policy discussions. In states and state Medicaid programs that have not adopted telehealth-supportive legislation or kept up with telemedicine policy changes in Medicare during the pandemic [27], there is a real threat to the financial sustainability of PCP telemedicine [28]. Specifically, if the majority of payers for patients within a pediatric primary care practice do not provide coverage at parity for telemedicine while the child is at home, then primary care clinicians may not be able to continue offering telemedicine to their patient panels [28]. As of June to August 2021, 63% of pediatricians reported that they were continuing to use telemedicine [29]; that number could rise or fall further depending on the ability of payers to signal and provide ongoing support for this modality of care. PCPs with concerns that patients may just as readily seek telemedicine care elsewhere may wish to take note of these results indicating that families value the continuity, pediatric expertise, and access to medical records of telemedicine through primary care practices offer.

While our data suggest that parents have the highest expectations for in-person primary care, it should be noted that parents’ expectations of PCP telemedicine approach expectations of in-person primary care for items in dimensions related to quality and even surpass in-person primary care for items related to access. In terms of virtual care options, our data suggest parents may preferentially choose PCP telemedicine for their children over DTC telemedicine, which is supported by the observation that the growth in telemedicine volume for children during the pandemic occurred almost entirely through PCP telemedicine rather than telemedicine-only providers [2]. PCP telemedicine also carries more positive expectations than urgent care or ED for access, quality, and parental self-efficacy dimensions. Thus, maintaining PCP telemedicine as an option may help families choose lower-cost options of care and maintain continuity. PCPs and health systems may also wish to ensure that patients can readily recognize and electronically engage PCP telemedicine, to ensure parents are connecting with the care and the providers that they desire.

Limitations include that results from qualitative interviews may not be generalizable to the population as this sample may over- or underrepresent certain populations when seeking care, such as female caregivers. Our research focuses specifically on how parents perceive telemedicine use in the context of seeking care for ARTI symptoms, and we note that these expectations could vary for other conditions. Interviews were conducted between April and July 2021, during a time when care-seeking decisions may have been influenced by the ongoing COVID-19 pandemic. However, the survey results were fielded in February 2022, a time when COVID-19 vaccines were available and parents had potentially 2 years of experience with telemedicine. We note also that while we had high rates of completion among those screened and determined to be eligible (1206/1297, 93%), there was a sizable number of nonresponders to the initial invitation to complete the screener, which may bias results.

In conclusion, in this mixed methods analysis of parent perceptions of telemedicine when approaching ARTI care-seeking decisions, parents expressed positive assessments of telemedicine accessibility while also voicing more concerns about telemedicine quality in commercial DTC models compared with primary care–based telemedicine. Future work is needed to help support families in making care-seeking decisions when their children are sick, by both supporting family decision-making and aligning in-person and telemedicine care options with child needs and family expectations.

## Appendix

Multimedia Appendix 1 Representative quotes from parent interviews (n=40) related to system-level dimensions.

Multimedia Appendix 2 Representative quotes from parent participants (n=40) related to parent or child factor dimensions.

Multimedia Appendix 3 Results from the survey (n=1206) illustrating the percentage that caregivers “always” or “often” expect the factors listed relating to expected accessibility, affordability, and quality at each of 5 sites (in-person PCP, ED, urgent care, PCP TM, DTCTM). DTCTM: direct-to-consumer telemedicine; ED: emergency department; PCP: primary care provider; PCP TM: primary care provider telemedicine.

Multimedia Appendix 4 Results from the survey (n=1206) illustrating the percentage that caregivers “always” or “often” expect the factors listed relating to child and parent-level factors at each of 5 sites (in-person PCP, ED, urgent care, PCP TM, DTCTM). DTCTM: direct-to-consumer telemedicine; ED: emergency department; PCP: primary care provider; PCP TM: primary care provider telemedicine.

## Abbreviations

ARTI: acute respiratory tract infection
DTC: direct-to-consumer
ED: emergency department
IRB: institutional review board
PCP: primary care provider

## Data Availability

Deidentified data will be made available on request from authors with appropriate institutional review board and data use agreements in place.

## References

[1] Schweiberger K, Hoberman A, Iagnemma J, Schoemer P, Squire J, Taormina J, Wolfson D, Ray KN (2020). Practice-level variation in telemedicine use in a pediatric primary care network during the COVID-19 pandemic: retrospective analysis and survey study. J Med Internet Res.

[2] Ray KN, Wittman SR, Yabes JG, Sabik LM, Hoberman A, Mehrotra A (2023). Telemedicine visits to children during the pandemic: practice-based telemedicine versus telemedicine-only providers. Acad Pediatr.

[3] Fiks AG, Frintner MP, Gottschlich EA, Ray KN (2022). Pediatricians' experiences with telehealth in 2021. Pediatrics.

[4] Ray KN, Shi Z, Poon SJ, Uscher-Pines L, Mehrotra A (2019). Use of commercial direct-to-consumer telemedicine by children. Acad Pediatr.

[5] Pooni R, Pageler NM, Sandborg C, Lee T (2022). Pediatric subspecialty telemedicine use from the patient and provider perspective. Pediatr Res.

[6] Katz SE, Spencer P, Stroebel C, Harnack L, Kastner J, Banerjee R (2021). Patient and provider perspectives on pediatric telemedicine during the COVID-19 pandemic. Telemed Rep.

[7] Kodjebacheva GD, Culinski T, Kawser B, Coffer K (2023). Satisfaction with telehealth services compared with nontelehealth services among pediatric patients and their caregivers: systematic review of the literature. JMIR Pediatr Parent.

[8] Kodjebacheva GD, Tang C, Groesbeck F, Walker L, Woodworth J, Schindler-Ruwisch J (2023). Telehealth use in pediatric care during the COVID-19 pandemic: a qualitative study on the perspectives of caregivers. Children (Basel).

[9] Russo L, Campagna I, Ferretti B, Agricola E, Pandolfi E, Carloni E, D'Ambrosio A, Gesualdo F, Tozzi AE (2017). What drives attitude towards telemedicine among families of pediatric patients? a survey. BMC Pediatr.

[10] Fischer SH, Predmore Z, Roth E, Uscher-Pines L, Baird M, Breslau J (2022). Use of and willingness to use video telehealth through the COVID-19 pandemic. Health Aff (Millwood).

[11] Ray KN, Shi Z, Ganguli I, Rao A, Orav EJ, Mehrotra A (2020). Trends in pediatric primary care visits among commercially insured US children, 2008-2016. JAMA Pediatr.

[12] Pines JM, Zocchi MS, Black BS, Carlson JN, Celedon P, Moghtaderi A, Venkat A (2021). Characterizing pediatric emergency department visits during the COVID-19 pandemic. Am J Emerg Med.

[13] Haddadin Z, Schuster JE, Spieker AJ, Rahman H, Blozinski A, Stewart L, Campbell AP, Lively JY, Michaels MG, Williams JV, Boom JA, Sahni LC, Staat M, McNeal M, Selvarangan R, Harrison CJ, Weinberg GA, Szilagyi PG, Englund JA, Klein EJ, Curns AT, Rha B, Langley GE, Hall AJ, Patel MM, Halasa NB (2021). Acute respiratory illnesses in children in the SARS-CoV-2 pandemic: prospective multicenter study. Pediatrics.

[14] Schroeder AR, Dahlen A, Purington N, Alvarez F, Brooks R, Destino L, Madduri G, Wang M, Coon ER (2022). Healthcare utilization in children across the care continuum during the COVID-19 pandemic. PLoS One.

[15] Schweiberger K, Patel SY, Mehrotra A, Ray KN (2021). Trends in pediatric primary care visits during the coronavirus disease of 2019 pandemic. Acad Pediatr.

[16] Nicholson E, McDonnell T, De Brún A, Barrett M, Bury G, Collins C, Hensey C, McAuliffe E (2020). Factors that influence family and parental preferences and decision making for unscheduled paediatric healthcare—systematic review. BMC Health Serv Res.

[17] Uscher-Pines L, Pines J, Kellermann A, Gillen E, Mehrotra A (2013). Emergency department visits for nonurgent conditions: systematic literature review. Am J Manag Care.

[18] Turnbull J, McKenna G, Prichard J, Rogers A, Crouch R, Lennon A, Pope C (2019). Sense-making strategies and help-seeking behaviours associated with urgent care services: a mixed-methods study. Health Serv Deliv Res.

[19] Burns SK, Krishnamurti T, Doan TT, Kahn JM, Ray KN (2023). Parent care-seeking decisions for pediatric acute respiratory tract infections in the United States: a mental models approach. Acad Pediatr.

[20] Watson G, Pickard L, Williams B, Hargreaves D, Blair M (2021). 'Do I, don't I?' a qualitative study addressing parental perceptions about seeking healthcare during the COVID-19 pandemic. Arch Dis Child.

[21] Ray KN, Wittman SR, Burns S, Doan TT, Schweiberger KA, Yabes JG, Hanmer J, Krishnamurti T (2023). Parent-reported use of pediatric primary care telemedicine: survey study. J Med Internet Res.

[22] O’Brien BC, Harris IB, Beckman TJ, Reed DA, Cook DA (2014). Standards for reporting qualitative research: a synthesis of recommendations. Acad Med.

[23] (2022). Panel design. NORC, AmeriSpeak.

[24] Bureau U Current population survey. United States Census Bureau.

[25] Rosenstock IM, Strecher VJ, Becker MH (1988). Social learning theory and the health belief model. Health Educ Q.

[26] Levesque JF, Harris MF, Russell G (2013). Patient-centred access to health care: conceptualising access at the interface of health systems and populations. Int J Equity Health.

[27] Center for Connected Health Policy (2023). State telehealth laws and reimbursement policies report, fall 2023. The National Telehealth Policy Resource Center.

[28] Ray KN, Keller D (2022). Telehealth and pediatric care: policy to optimize access, outcomes, and equity. Pediatr Res.

[29] Ralston SL, Lieberthal AS, Meissner HC, Alverson BK, Baley JE, Gadomski AM, Johnson DW, Light MJ, Maraqa NF, Mendonca EA, Phelan KJ, Zorc JJ, Stanko-Lopp D, Brown MA, Nathanson I, Rosenblum E, Sayles S, Hernandez-Cancio S (2014). Clinical practice guideline: the diagnosis, management, and prevention of bronchiolitis. Pediatrics.

