# Supplementary material for: Parent Perceptions of Telemedicine for Acute Pediatric Respiratory Tract Infections: Sequential Mixed Methods Study
Source: JMIR Pediatr Parent. 2024 Jan 16;7:e49170. doi: 10.2196/49170 (PMC10828946; doi:10.2196/49170)
Supplement: Multimedia Appendix 1 [file pediatrics_v7i1e49170_app1.docx]

**Multimedia Appendix 1.** Representative quotes from parent interviews (n=40) related to system-level dimensions.

| **Dimension** | **Factor** | **Quote** |
| --- | --- | --- |
| **Expected Accessibility**  **of the Site** | Temporally Accessible | So I would see it as like, “Well, I don’t really want to get a babysitter and take the other one in to the doctor’s, but I guess I’ll do telemedicine. That seems like less work.” (parent 38) |
|  | Geographically Accessible | We’re about a half hour away from the hospital, so anything we can do virtual we can—we do it, just because it’s easier. (parent 39) |
|  | Convenience Maximized | When you go into the hospital, it’s always like we’ve got to go to the waiting room, and then it’s a long wait… it always takes like four hours opposed to being at home on a computer, it takes 30 minutes or less, and you get all the information that you can get. (parent 20) |
|  | Telemedicine as a site of last resort | But if it’s not possible [to be seen in person]… I won’t decline it and say, “No, if it’s not in person, then I don’t want your input.” Like, I would still—if I knew that it was a last resort, then it’s fine… If I have the option, I would rather, like, set an appointment, or go see the professional myself. (parent 22) |
|  | Digitally Accessible | **Digital Accessibility:** Unless we use the phone… the only like computers we have at home are our like work computers. We don’t like have personal computers or a desktop computer, and I don’t necessarily have my work computer on me at home all the time, so…you know, depending on like where we were, I mean, that would probably make [using telemedicine] difficult. (parent 06) |
|  |  | **Digital Literacy:** So I guess it depends on how [telemedicine platform is] set up. If it’s more like a direct line… there’s like, an operator that’s on call 24/7...that will be probably easier to use, and I would use that platform to do so. It just depends how the set up is. And if the setup’s a little bit too much, it’d probably be easier for me just to take her to a facility. (parent 22) |
| **Expected Affordability**  **of the Site** | Cost Reduction | **Cost Reduction:** I guess because you’re not getting the...the in-person evaluation. That’s probably why I’m like, “maybe [telemedicine] should [cost] a little less. But you did give your advice.” They were there. They were able to log in and show your undivided attention, and all of that. And you still was able to probably get what you need—it just wasn’t a physical evaluation. (parent 22) |
|  |  | **Insurance Coverage:** My understanding was always that it’s [a telemedicine visit] just billed… like a doctor’s visit and covered the same way, so. I don’t—I don’t think it would make any difference if I went into the office—for my insurance plan. (parent 11) |
|  |  | **Likelihood of follow-up costs:** If it’s the instance where… the issue does [not] get resolved and I might have to go back, and then have another copay, like I wouldn’t want telehealth, I would just want to go into the office. So that I could just get everything solved at once. (parent 04) |
|  |  | **Out-of-Pocket costs:** I mean, what would the cost be [for doing a telemedicine visit]? I mean, if you were connecting to somebody at your doctor’s office, would it be just a sick copay, would it be a different thing? I mean… I’m obviously not going to do something if it’s way more expensive than what we would normally do. (parent 06) |
| **Expected Quality**  **of the Clinician** | Continuity of Care | Telemedicine is not really something I’m totally in favor for, even though I’m more, “Get with the times,” you know, but I’m more set in stone, like, go to the doctor. They can run tests. They can see you. They know. They can ask you how your kids acts, versus doing the telemedicine thing which might not even be with your doctor. (parent 28) |
|  | Trustworthy Care | Like you are getting a—a less-then-ideal…doctor’s visit by going through, you know, John Doe Doctor online than you are going to your pediatrician that you picked because you like them, and you trust them, and they know your child. (parent 11) |
|  | Parental Reassurance | Something about just the, the...interaction [in-person], I guess, between you and the doctor. Or you and the nurse, and the personal...it just feels more personal than if it was telemedicine. (parent 17) |
|  | Pediatric Expertise | I think it [DTCTM] would be fine. Like it wouldn’t bother me that they weren’t a part of my pediatrician’s office, ‘cause that’s kind of what you get when you go to urgent care. Like you said, it’s whoever is working at the time. So I don’t. I think that would be fine. I would be fine with it. (parent 41) |
| **Expected Quality**  **of the Site** | Ease of Assessment | If it’s ear infection type things, like I can tell them, “Oh, she’s pulling at her ears,” but they could be itchy, they could be filled with wax, like it could be swimmer’s ear; but until—like unless they look in her ear to see what’s happening, I feel like they can’t really do much. Like I can tell them what they’re doing, but they can’t see anything [on telemedicine]. (parent 41) |
|  | Receptivity of Care Delivery | I do like the idea [of telemedicine], I think people just need to get a little more used to it. Like I’ve never done it before, but I wouldn’t be opposed to doing it. (parent 10) |
|  | Safe Care | They might also pick other stuff up when they go to the doctor’s office. So [telemedicine] might just be an ease, so you’re not exposing yourself to other things. (parent 39) |
|  | Clinical Resources at Site | And, obviously, her check-ups, we can do a couple on telemed, but they need to draw blood to check her numbers—her hemoglobin, so that we can’t do. (parent 24) |
|  | Child’s Comfort Level | The only challenge [using telemedicine] I can think of is if, like, it’s you and a sick child, and that sick child is screaming or upset or whatever, like, it can be really hard to have a, like, thoughtful conversation about what’s going on if you’re trying to like corral the child at the same time… (parent 08) |
