# Supplementary material for: Parent Perceptions of Telemedicine for Acute Pediatric Respiratory Tract Infections: Sequential Mixed Methods Study
Source: JMIR Pediatr Parent. 2024 Jan 16;7:e49170. doi: 10.2196/49170 (PMC10828946; doi:10.2196/49170)
Supplement: Multimedia Appendix 2 [file pediatrics_v7i1e49170_app2.docx]

**Multimedia Appendix 2.** Representative quotes from parent participants (n=40) related to parent/child factor dimensions

| **Dimension** | **Factor** | **Quote** |
| --- | --- | --- |
| **Perceived**  **Severity of Illness of the Child** | Symptom Severity | If they’re really sick, I would feel more comfortable if they’re actually seen in person. (parent 05) |
|  | Indicator of Serious Illness | I feel like stuff that might be more serious shouldn’t be evaluated on telehealth. ‘Cause they’re just going to tell you to come in anyway. (parent 26) |
|  | Symptom Persistence | Yeah, ‘cause I feel like they’re going to tell you [on telemedicine] if they say, “Okay, give it two more days, or 24 hours, and if the fever hasn’t broken, or if it’s still above X, Y, Z, then, you know, call us back, or…you know, come in to the office.” (parent 40) |
|  | Symptom Complexity | I think if it’s just like a…kind of like a normal cold, like slight cough, congestion, and maybe like a low-grade fever for like the first day or two, and they’re not complaining of like their ear pain or anything actually bothering them, I think [telemedicine is] a good option. (parent 34) |
|  | Illness-Associated Demeanor | I never have [used telemedicine when daughter is sick] only because my daughter is very hyper, so it’s really hard to determine—like she doesn’t get lethargic as much, even if she is, she still moves around and she’s still active, because she doesn’t understand that she’s sick and she needs to lay down and relax her body. So she still acts normal. So it’s really hard for me to tell sometimes. (parent 20) |
| **Perceived Susceptibility of the Child** | Community-Based Exposure | I prefer contact so I can be like hands-on, but in a situation where I needed to use telemedicine, or I, you know, had no other choice right now because of the pandemic… that we had to use telemedicine, I would totally use it… (parent 27) |
|  | Child Age | So you can tell the doctor what you think they’re [baby] feeling, like kind of interpret how they’re feeling, but you don’t know for sure. And the doctor really has the ability to...like, I think just touch is a big factor in diagnosing an illness. You know, like, I said, feeling their glands, making sure their tummies are soft, and just listening to their heart and their lungs; you can’t do that in telemedicine. (parent 18) |
|  | Child Vulnerability to Illness | Anytime she doesn’t sleep, she normally always has a double ear infection. So, like if it was a different child maybe [would use telemedicine], but like me knowing her history, I would have just brought her in. (parent 34) |
|  | Child Medical Complexity | I mean like in our situation… it [telemedicine] wouldn’t be helpful if… if it’s more of like an urgent care and you’re having to explain yourself, it wouldn’t be helpful in that way. (parent 02) |
| **Perceived Self-Efficacy of the Parent** | Achieving Goal of Visit | I thought I was gonna get some sort of steroid or something, but they just basically said, “Oh, go home and stand outside in the cold,” which that could’ve definitely just been… something that was told over the computer or even over the phone. (parent 14) |
|  | Parent Health Literacy | If it’s something that I’m pretty confident and I know what it is and I can explain what the symptoms are, like, that, fine, great. That [being seen on telemedicine] would save me a lot of time and a lot of hassle and a lot of taking their sick butts outside. (parent 09) |
|  | Antibiotic Expectations | And I think they sent her like an antibiotic, or something …they could’ve—you know, on telemedicine they would have been able to tell that, if they would have heard her cough, so I feel like that would’ve really saved us a visit… (parent 26) |
|  | Easing Uncertainty | There have been times where… I’ve kind of been on the fence about, you know, she’s been sick for a little while and doesn’t seem to be any better and maybe it’s like… later in the evening and I said to myself, well, I’ll just wait and see how she is in the morning. (parent 04) |
|  | Worry | **Worry:** I mean, I feel like it [telemedicine] could be helpful in certain situations, but emergency services… See, I’m a panic-er, so I’m not gonna wait around on telemedicine—I’m gonna run to the emergency room. (parent 37) |
|  |  | **Worry about Privacy:** I mean, I figure if you’re talking to a provider [on telemedicine], they’re gonna be in an office, or an empty clinic room, or something like that, and you’re not gonna...there’s not gonna be any less privacy, I guess, than if you were actually there. (parent 18) |
|  |  | **Worry about Home Environment:** Oh, I definitely think that’s [provider seeing in home] a huge benefit, where they kind of get a…even if it’s just a little picture, like it’s still a little idea of what the home environment is like, and I think that really goes a long way in like completing the puzzle on this child that they’re caring for. (parent 38) |
|  |  | **Worry about Equity:** Families that maybe are lower in income, that don’t have a transportation method—telehealth will be revolutionary, that’ll be perfect. (parent 21) |
|  |  | **Worry about Paternalism:** I think if anything, it [telemedicine experience] was like neutral to good. Like, it was a fine experience and, you know, it’s—both times, the physicians were not… in a tie and a button-down and a coat; like, they were just kind of in, like, a t-shirt, so it was like, “Wow, this is relaxed. It’s kind of nice.” Like, you’re maybe slightly more approachable in some way. (parent 07) |
