# Supplementary figures and images for: Parent Perceptions of Telemedicine for Acute Pediatric Respiratory Tract Infections: Sequential Mixed Methods Study
Source: JMIR Pediatr Parent. 2024 Jan 16;7:e49170. doi: 10.2196/49170 (PMC10828946; doi:10.2196/49170)

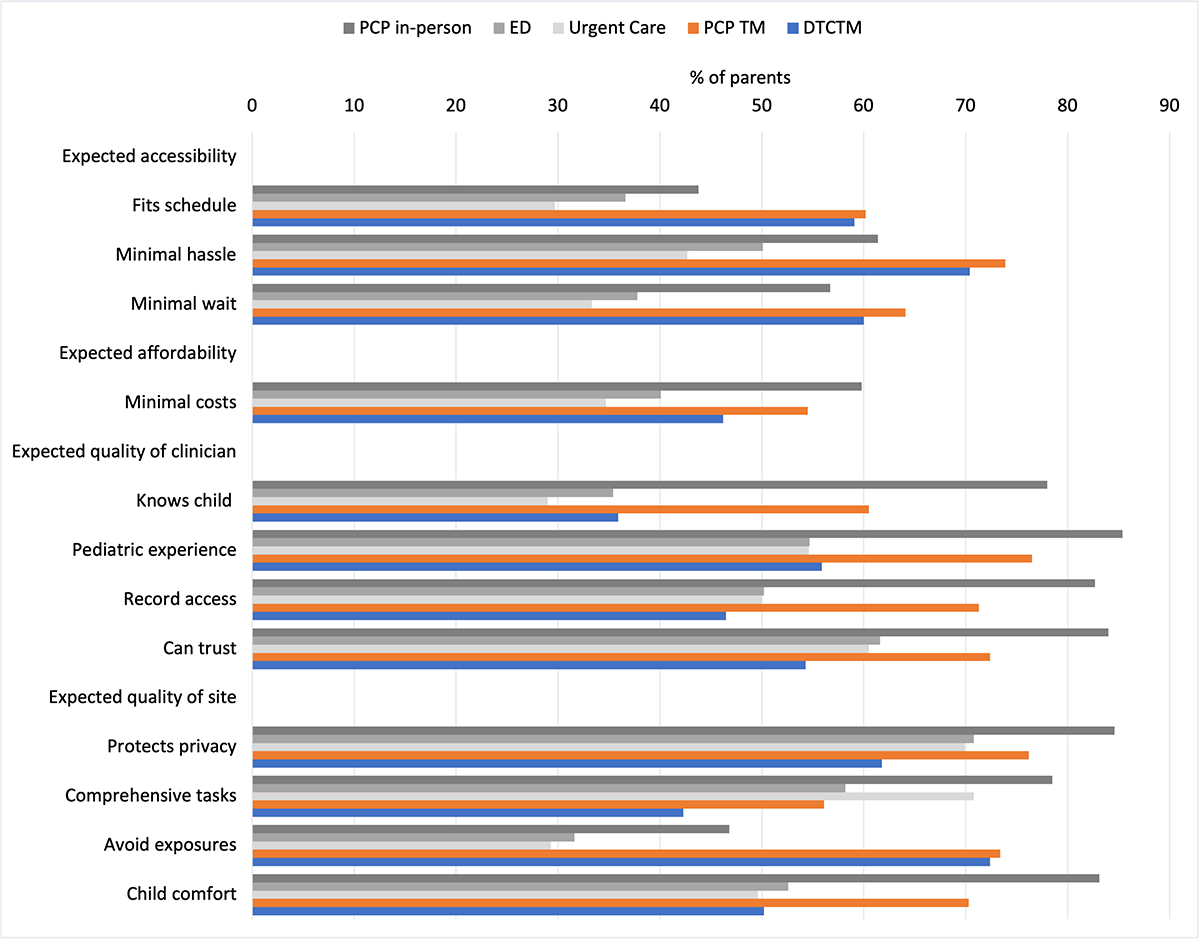

Supplement: Multimedia Appendix 3 [file pediatrics_v7i1e49170_app3.png]

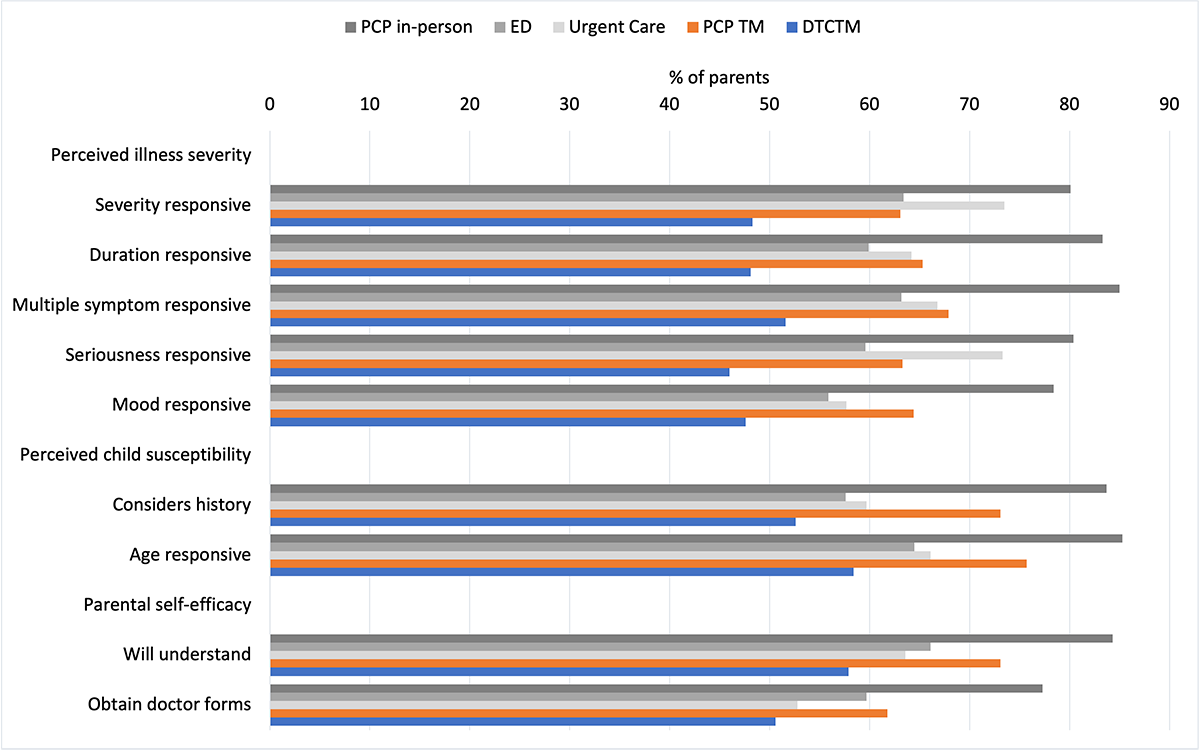

Supplement: Multimedia Appendix 4 [file pediatrics_v7i1e49170_app4.png]
